# Start of a New Era: Management of Non-Clear Cell Renal Cell Carcinoma in 2022

**DOI:** 10.1007/s11912-022-01269-1

**Published:** 2022-04-19

**Authors:** Benjamin L. Maughan

**Affiliations:** Huntsman Cancer Institute, University of Utah, 2000 Circle of Hope Drive, Room HCI S 5617, Salt Lake City, UT 84112 USA

**Keywords:** Chromophobe, Papillary, Collecting duct, Non-clear cell, Kidney cancer, Immune therapy, Tyrosine kinase inhibitor, Unclassified, Translocation

## Abstract

**Purpose of Review:**

Historically, kidney cancer was diagnosed as either clear cell renal carcinoma (ccRCC) or non-clear cell renal carcinoma (nccRCC). With further research into the pathophysiology of nccRCC, multiple distinct subtypes have emerged creating distinct diagnosis, such as papillary renal cell carcinoma (PRCC), chromophobe renal cell carcinoma (crRCC), or unclassified carcinoma (cRCC). Many other kidney cancer subtypes are now included in the WHO classification system.

**Recent Findings:**

The prognosis for each of the more frequently diagnosed types is discussed here along with treatment recommendations. The available clinical trial results and salient retrospective studies of each subtype are reviewed here to guide clinicians on the optimal treatment selection for patients with these rare histologic types or RCC.

**Summary:**

Many nccRCC types are now recognized and each has unique molecular drivers which are different than ccRCC. The optimal treatment strategy is different for each subtype. The prognosis also differs based on the histology.

## Introduction

Oncologists are anticipated to treat more patients with renal cell carcinoma now compared to previous generations of physicians. The American Cancer Society estimates that the incidence of renal cell carcinoma is increasing steadily, with an estimated 7.1 cases per 100,000 people in 1975 in contrast to an estimated 16.1 cases per 100,000 people in 2017. In contrast, some other cancers have noted decreasing trends such as colorectal, lung, and stomach cancers [1]. Fortunately, there has been a significant expansion of our understanding of renal cell carcinoma and treatment paradigms for patients with renal cell carcinoma.

The most recent WHO classification system for renal cell carcinoma published in 2016 [2] includes both aggressive and indolent cancers: clear cell renal cell carcinoma, multilocular cystic renal neoplasm of low malignant potential, papillary renal cell carcinoma (PRCC), fumarate-hydratase deficient renal cell carcinoma (fhRCC), chromophobe renal cell carcinoma (chRCC), collecting duct carcinoma (CDC), renal medullary carcinoma (RMC), MiT family translocation renal cell carcinomas (tRCC), succinate dehydrogenase deficient renal cell carcinoma (SDDRCC), mucinous tubular and spindle cell carcinoma, tubulocystic renal cell carcinoma, acquired cystic disease–associated renal cell carcinoma, clear cell papillary renal cell carcinoma, unclassified renal cell carcinoma (uRCC), papillary adenoma, and oncocytoma among many others. This review will focus on the more aggressive subtypes including PRCC, fhRCC, chRCC, CDR, RMC, tRCC, and uRCC. This diagnostic framework is continually evolving with increased understanding of the biologic underpinnings of these malignancies. It is anticipated that an updated classification will be published again possibly in 2022 or later.

**Fig. 1:**
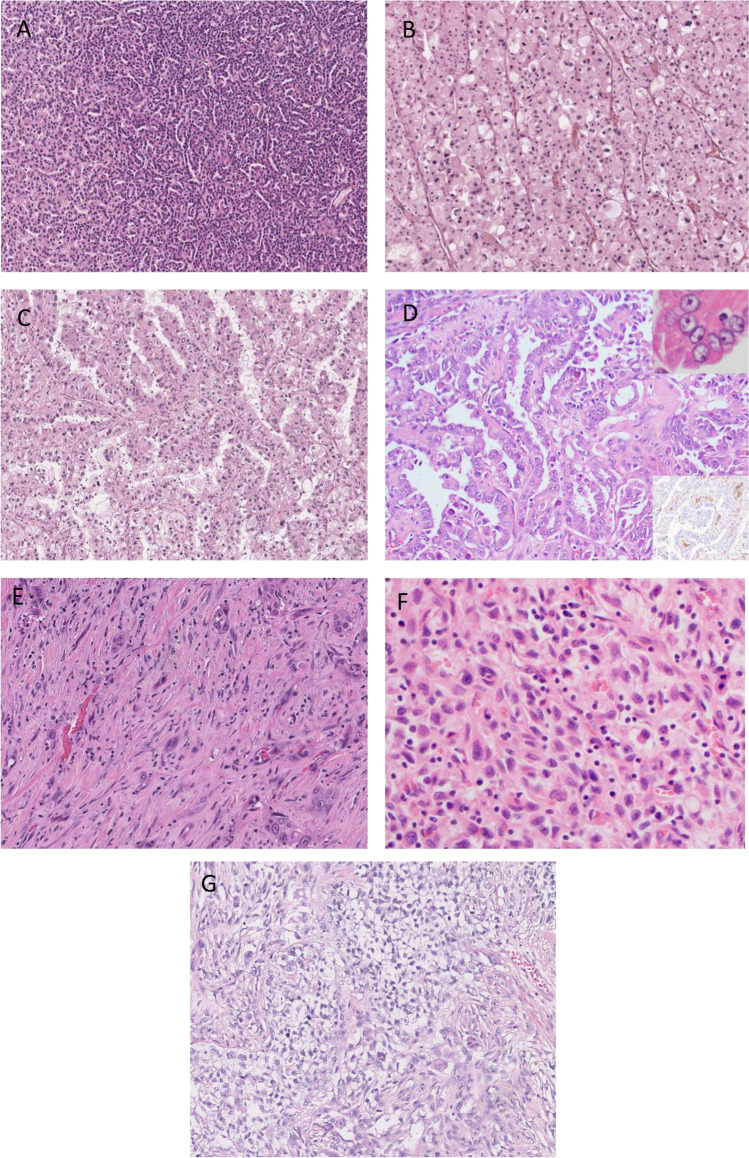
Photomicrographs of selected non-clear cell renal carcinomas. **A** Papillary renal cell carcinoma shows a conspicuous papillary architecture (H&E, 100 ×). **B** Chromophobe renal cell carcinoma: tumor cells with plant-like cell borders, rasinoid nuclei, and perinuclear halos (H&E, 100 ×). **C** TFE3 rearranged renal cell carcinoma: a papillary architecture cell with abundant clear and eosinophilic cytoplasm characterizes this subtype of renal cell carcinoma (H&E, 100 ×). Inset: fluorescence in situ hybridization for TFE3 shows a break-apart signal. **D** Fumarate hydratase–deficient renal cell carcinoma: a papillary architecture with moderate cytoplasmic eosinophilic change is seen (H&E, 100 ×). The top inset demonstrates characteristic nuclei with prominent eosinophilic nucleoli and perinucleolar halos. The bottom inset shows loss of fumarate hydratase identified on a fumarate hydratase immunohistochemical stain. **E** Collecting duct carcinoma: infiltrating atypical glands in a background of desmoplastic stroma characterize this renal cell carcinoma (H&E, 100 ×). **F** Unclassified renal cell carcinoma: the image depicts a renal cell carcinoma with sarcomatoid morphology (H&E, 100 ×). Ancillary work up was inconclusive for a known RCC subtype. **G** Renal medullary carcinoma: this rare subtype of renal cell carcinoma shows poorly differentiated glands in a reticular myxoid background (H&E, 100 ×)

Despite this diverse diagnostic categorization, historically, renal cell carcinoma was clinically treated as two disease entities: clear cell renal carcinoma (ccRCC) and non-clear cell renal carcinoma (nccRCC). Clinical trials included patients with any of these nccRCC diagnosis and also collated all the patients when reporting results. This facilitated clinical trial accrual but limited the capacity to advance our understanding of each subtype. Also the clinical trials were generally based on testing therapies proven in ccRCC without consideration of the unique biology for each histology/molecular diagnosis. The ASPEN [3] and ESPN [4] trials are examples of this approach. More recently, clinical trials are separating patients by diagnosis in the inclusion criteria and/or analysis leading to more therapeutic advances (see Fig. 1).

### Papillary Renal Cell Carcinoma

PRCC is further subclassified histologically (and increasingly by molecular characteristics) into type 1 PRCC and type 2 PRCC [5, 6]. Molecular alterations to *MET* are frequently found in type 1 including mutations and amplification with duplication of chromosome 7, the location of *MET*. Other molecular alterations can also be observed including *SETD2*, *NF2*, *KDM6A*, *TERT*, and *SMARCB1* as well as chromosome gains in other chromosomes. Alterations to *MET* are observed in over 80% of patients [7••]. Type 2 includes a more diverse group of molecular drivers including hypermethylation of *CDKN2A* resulting in gene silencing, mutations to chromatin remodeling genes (e.g., *PBRM1, SETD2, BAP1*), *TFE3* fusions, and increased expression of the NRF2–antioxidant response element pathway [7••].

Generally, type 1 PRCC presents with more high risk features and frequently has a worse prognosis relative to type 2 PRCC. Currently, the diagnostic criteria between these two subtypes are histologically defined. Not surprisingly, there are frequently identified cases that share similar histologic features of both type 1 and type 2 [8]. The pathologic distinction between type 1 and type 2 PRCC can be challenging with frequent discordance between pathologists. Many centers prefer to classify all PRCC together and avoid subtyping due to this diagnostic inconsistency.

Tyrosine kinase inhibitors (TKI) are frequently effective for these cancers. A multi-institution retrospective study suggests that cabozantinib (a multi-targeted TKI including MET) is highly effective for this disease [9]. Recently, the S1500 clinical trial was published establishing a standard of care treatment based on prospective clinical trial data. This cooperative group clinical trial randomized patients 1:1:1:1 to the standard of care at that time, sunitinib (multi-targeted TKI including VEGF), compared separately with each of three investigational arms: cabozantinib, savolitinib (a more selective MET inhibitor), and crizotinib (ALK and MET antagonist) [10••]. In total, 152 patients were enrolled: 48 to sunitinib, 46 to cabozantinib, 28 to crizotinib, and 30 to savolitinib. The latter two arms enrolled fewer patients because of inferior results compared with sunitinib at the time of the interim futility analysis resulting in early closure. The median progression-free survival (PFS), the primary endpoint, was 5.6 months (95% *CI* 2.9–6.7), 9.0 months (95% *CI* 5.6–-12.4), 2.8 months (95% *CI* 2.6–3.6), and 3.0 months (95% *CI* 2.8–7.2), respectively. This is the first randomized clinical trial specifically in PRCC and establishes cabozantinib, the current standard of care for first-line treatment of PRCC.

*MET* alterations are not the only molecular drivers of PRCC as seen from the S1500 clinical trial where savolitinib, a *MET*–targeted therapy, was inferior to sunitinib. This difference was observed in patients with both type 1 and type II histology. Tumors with *MET* alteration do appear sensitive to savolitinib though. The SAVIOR clinical trial was a randomized phase III clinical trial comparing savolitinib versus sunitinib in PRCC patients with *MET* alterations [11]. The trial was closed early due to slow accrual. In total, 60 patients were randomized (*n* = 33 savolitinib, *n* = 27 sunitinib). The median PFS was 7.0 months and 5.6 months, respectively (*p* = 0.31). *MET* status was defined as any of chromosome 7 gain, *MET* amplification, *MET* kinase domain variations, or HGF amplification. Savolitinib is not approved for the treatment of RCC outside of China.

Immune therapy using PD-1/PD-L1 checkpoint inhibitors has also been tested in PRCC with apparent clinical activity in some patients. KEYNOTE-427 was the first clinical trial reported using immune checkpoint inhibitors in treatment-naïve patients with nccRCC. This clinical trial enrolled patients into two cohorts, ccRCC (*n* = 110) [12] and nccRCC (*n* = 165) [13•]. All patients received open-label pembrolizumab 200 mg every 3 weeks for ≤ 24 months. The results of the nccRCC cohort were reported for each histology separately including PRCC (71.5%), CRCC (12.7%), and unclassified (15.8%). For PRCC, the objective response rate (ORR) was 28.8%, similar to what has been observed with first-line single-agent checkpoint inhibitors in the ccRCC population, 36.5% with pembrolizumab [12]. The ORR is also similar to that observed with second-line checkpoint inhibitors in the ccRCC population, 23% for nivolumab [14]. The CheckMate-374 study is a phase IV clinical trial that evaluated nivolumab in nccRCC [15•]. A total of forty-four patients were enrolled. Patients could have received up to 3 prior lines of therapy. Most patients had PRCC (*n* = 24), followed by chRCC (*n* = 7), uRCC (*n* = 8), tRCC (*n* = 2), CDC (*n* = 1), RMC (*n* = 1), and not reported (*n* = 1). No complete responses (CR) were observed in PRCC patients and there were only 2 partial responses (PR) for an ORR of 8.3%. Nine patients with PRCC had stable disease (SD) for a disease control rate (DCR, CR + PR + SD) of 45%. The median duration of response was 10 months.

Based on the results of TKI/IO therapy in ccRCC, some phase II clinical trials have been conducted in PRCC. Savolitinib combined with durvalumab (PD-L1 antagonist) was conducted in a phase 1 dose escalation study (*n* = 41) [16]. The combination was safe with an observed response rate of 27% with a median PFS of only 3.3 months. The ORR was higher in patients with *MET* alterations (40%). Separately, cabozantinib plus nivolumab (PD-1 antagonist) was explored in all nccRCC [17]. Two separate cohorts were enrolled: cohort 1 enrolled PRCC (*n* = 33), translocation (*n* = 2), and unclassified (n = 5); cohort 2 enrolled only chromophobe (*n* = 7). No CR were observed in cohort 1. The ORR was 47% and only 1 patient had progressive disease (PD). The median duration of response was 13.6 months for a 12-month PFS of 52.8%.

Based on the results of the S1500 clinical trial, the recommended first-line therapy is cabozantinib. Emerging evidence suggests that TKI-IO–based combinations might be highly active in PRCC with trials ongoing to formally evaluate this approach. For instance, SWOG is opening S2200 (PAPMET2) comparing cabozantinib versus cabozantinib plus atezolizumab as first-line therapy in patients with PRCC. This is anticipated to open in 2022.

### Fumarate Hydratase–Deficient Renal Cell Carcinoma

Fumarate hydratase–deficient renal cell carcinoma (fhRCC) is characterized by loss of the *FH* gene and was first identified as part of the inherited disease hereditary leiomyomatosis and renal cell cancer (HLRCC) in which patients have a germline loss of *FH* function. These patients develop benign uterine and cutaneous leiomyomas as well as very aggressive RCC cancers. More recently, this disease has been observed to develop in consequence to germline as well as somatic events. Patients with metastatic disease have a very poor prognosis with many patients presenting with metastatic disease at the time of initial diagnosis.

Historically, FHRCC has been included histologically as PRCC type 2, but given the specific molecular characterization and poor prognosis, it is more recently categorized separately. Unique surveillance strategies as well as systemic treatment recommendations exist for this disease.

A retrospective analysis of HFRCC patients was recently reported [18]. This included twenty-four patients. The response rate to PD-1/PD-L1–based therapy was only 18% with a median time to treatment failure of only 2.5 months. TKI–based therapy had superior results with an ORR of 50% for cabozantinib and 63% for sunitinib. The median time to treatment failure was 11.6 months. The ORR to mTOR–based therapy was 0%.

The National Institute of Health conducted the first prospective clinical trial for fhRCC [19••]. Patients with fhRCC (*n* = 42) or sporadic PRCC (*n* = 41) were enrolled into separate cohorts. All patients were treated with bevacizumab plus erlotinib. The ORR for fhRCC was 64% and only 37% for PRCC. The median PFS was 21.1 months and 8.7 months, respectively. It is recommended to prioritize TKI–based therapy for these patients.

Immune therapy appears to have a limited role in FHRCC though these patients can have moderately durable responses to TKI–based therapy. Bevacizumab/erlotinib is the recommended first-line therapy followed by sequential TKI therapy at disease progression.

### Chromophobe Renal Cell Carcinoma

Chromophobe renal cell carcinoma (chRCC) has multiple molecular alterations including mutations to *PTEN* and *TP53*, whole chromosome loss, and frequent *TERT* gene rearrangements. These cancers appear to be highly dependent on oxidative phosphorylation through the Krebs cycle compared with normal renal tissue [20]. The prognosis for these patients is more favorable relative to other RCC types, with fewer patients having metastatic disease at diagnosis [2]. The IMDC group published survival data from a total of 10,105 patients by site of metastasis compared between ccRCC (92%), PRCC (7%), and chRCC (2%) [21]. Patients with chRCC have superior survival compared with ccRCC and PRCC regardless of the site of metastasis evaluated. Presence of lung metastasis was the only exception with chRCC having an inferior median OS (14.1 months [95% *CI*, 8.2–23.8 months] compared with ccRCC (25.1 months [24.1–26.0 months]; *P* < 0.001). The TCGA dataset was also used to compare the prognosis of chRCC to ccRCC and reported a similar favorable prognosis for chRCC relative to ccRCC [22].

There are no prospective clinical trials specifically in chRCC. ASPEN [3] and ESPN [4] are two randomized basket clinical trials of nccRCC that includes patients with chRCC. Both studies compared sunitinib versus everolimus (MTOR antagonist). Twelve and 16 patients with chRCC were enrolled on the ESPN and ASPEN clinical trials, respectively. In ASPEN, ten patients received sunitinib with a median PFS of 5.5 months (95% *CI* 3.2–19.7 months). Six patients were treated with everolimus with a median PFS of 11.4 months (95% *CI* 5.7 to 19.4 months). In ESPN, six patients were treated with sunitinib with a median PFS of 8.9 months and 6 patients were treated with everolimus though the median PFS was not reported.

Not all TKIs appear to have equal efficacy in this histology. For instance, two prospective single-arm clinical trials [23, 24] evaluated sorafenib in nccRCC and included a total of 32 (3 + 29, respectively) patients with chRCC. The ORR in the chRCC patients was < 5% combined between both studies. In contrast, sunitinib was tested in two separate single-arm prospective trials in nccRCC with a reported ORR of 33.3% (chRCC, *n* = 5) [25] and an ORR of 40% (chRCC, *n* = 5) [26•].

PD-L1 expression is lower [27] in these cancers as well as less immune infiltration [28] suggesting a poor response to checkpoint inhibitor therapy. Data from prospective studies confirms this observation. The KEYNOTE-427 trial, cohort-B included 21 patients with chRCC (12.7%) [13•]. The ORR to pembrolizumab was only 9.5% (95% *CI*, 1.2 to 30.4%) and the median PFS was only 3.9 months (95% *CI*, 2.6 to 6.9). The prospective clinical trial of cabozantinib plus nivolumab included a cohort of seven patients with chRCC [17]. This cohort was designed with a Simon two-stage stopping rule and was closed early due to a poor response rate. No patients with chRCC had a PR or CR.

The optimal therapy is yet to be determined for this disease. However, TKI therapy appears to have a higher response rate compared with checkpoint inhibitors and therefore it is recommended to prioritize TKI therapy over immune therapy at this time.

### Collecting Duct Carcinoma

Collecting duct carcinoma (CDC) is a very rare malignancy that is often metastatic at the time of diagnosis and portends a very poor prognosis [29, 30, 31•]. Morphologically, it is similar to RMC though it can be differentiated by molecular characteristics. It also shares some morphologic characteristics with urothelial carcinoma. CDC has retained INI1 though frequently has *HER2* amplification and mutated *SMARCB1*, *NF2*, and *SETD2* genes [32].

The largest reported CDC cohort included 577 patients identified through the National Cancer Database [31•]. Seventy percent of patients in this dataset had metastatic disease at diagnosis with a median OS of only 13.2 months.

Treatment typically involves cytoreductive nephrectomy with or without chemotherapy and radiation as this cancer is not responsive to most traditional RCC therapies such as sunitinib [26•]. In the National Cancer Database, the median survival was most prolonged with the combination of cytoreductive nephrectomy plus chemotherapy/radiation (9.9 months; 95% *CI* 7.6–12.1 months) compared with surgery alone (4.4 months; 95% *CI* 1.8–7.0 months) or chemotherapy/radiation alone (5.8 months; 95% *CI* 3.8–7.7 months) [31•].

Platinum chemotherapy is clinically active in this disease as shown in a prospective clinical trial testing cisplatin or carboplatin plus gemcitabine [33]. Twenty-three patients were enrolled. The ORR was 26% with a median PFS and OS of 7.1 months (95% *CI* 3.0–11.3) and 10.5 months (95% *CI* 3.8–17.1), respectively.

Surprisingly, cabozantinib appears to also have clinical activity. It was recently examined in a separate prospective clinical trial of 25 patients [34••]. The ORR was 35% with a disease control rate of 71%. The median follow-up was short however at only 8 months.

Currently, there is no optimally identified treatment for these patients though both platinum chemotherapy and cabozantinib can be considered. Further clinical trials are needed to improve the dismal prognosis for these patients.

### Renal Medullary Carcinoma

Renal medullary carcinoma (RMC) is very rare but almost exclusively diagnosed in patients with sickle cell trait or sickle cell anemia. It is notable for *SMARCB1* deletion resulting in the complete loss of the INI1 protein. The prognosis for these patients is very poor. No prospective clinical trials have been conducted specifically in this disease though some patients have been included in basket trials including nccRCC histology. These cancers also do not respond to typical TKI–based therapy [26•, 35].

Retrospective case series suggest that platinum chemotherapy can be effective. The largest case series published to date includes 52 patients [36•]. Fifty-four percent of patients were treated with a targeted therapy. Unfortunately, there were no objective responses observed. Chemotherapy resulted in an ORR of 29% with a median OS of 16.4 months for patients treated with chemotherapy plus nephrectomy compared with 7.0 months for chemotherapy alone. Most chemotherapy used included cisplatin or carboplatin (carboplatin/paclitaxel ± bevacizumab; gemcitabine/cisplatin ± bevacizumab; gemcitabine/doxorubicin ± bevacizumab; and dose-dense MVAC-methotrexate, vinblastine, doxorubicin, cisplatin).

### Translocation Carcinoma

Translocation carcinoma (tRCC) encompasses a diverse range of fusions that involve one of three genes: *TFE3*, *TFEB*, and *MITB*. The *TFE3* is located on Xp11.2; hence, the other name for this subgroup is Xp11 translocation carcinoma, though it should be noted that the term is not inclusive of all the translocations observed in this category. The morphology of tRCC is similar to ccRCC and PRCC making diagnosis purely on morphology and histology difficult. Currently, use of FISH probes specific for these translocations is the gold standard diagnostic test [37] though RNAseq and RT-PCR might be used more frequently in the future [38].

tRCC has a higher incidence in pediatrics compared with adult patients but is uncommon in any age range. The prognosis appears to be variable based on the specific translocation present with some retrospective data suggesting *TFEB* rearrangements have a more favorable prognosis compared with *TFE3* rearrangements. Most of these translocations result in aggressive biology and a poor prognosis [39–41].

Approximately, 10% of patients have high PD-L1 expression (≥ 5% tumor cell membrane staining), similar to PRCC [27]. The number of tumors evaluated in this study was low (50 patients PRCC; 10 patients tRCC) adding some uncertainty to the veracity of this conclusion. It is unclear if tRCC is sensitive to checkpoint inhibitor therapy despite a relatively frequent PD-L1 expression as there are no prospective studies specifically in this population. In the Checkmate-374 study testing nivolumab monotherapy in both ccRCC and nccRCC, a total of 2 patients with tRCC were enrolled. Both patients experienced PD as the best response to therapy [15•].

Some prospective basket trials of nccRCC have shown efficacy with TKI therapy. A study of 52 patients, 7 with tRCC, treated with axitinib after progression on temsirolimus reported an ORR of 57.1% (4/7 patients) and a disease control rate of 85.7% [25]. The median PFS with sunitinib and temsirolimus in the ESPN study which enrolled 7 patients with tRCC was 6.1 months (95% *CI* 6.0–8.8; *n* = 3) and 3.0 months (95% *CI* 1.3–NA, *n* = 4), respectively [4]. The ASPEN trial enrolled eight patients with tRCC but did not report the OS or PFS results for this group. No patients with tRCC achieved a CR or PR to either sunitinib or temsirolimus [3].

Retrospective reports have identified some responding patients to both checkpoint inhibitors and TKI therapy. The multi-institutional report of 43 patients with nccRCC treated with PD-1/PD-L1 therapy including three patients with tRCC was notable for one patient with a PR, one patient with SD, and one patient with PD [42]. A study evaluating treatment with any VEGF therapy in fifteen patients with tRCC reported an ORR of 20% (3/15), and a median PFS and OS of 7.1 months and 14.3 months, respectively [43]. Cabozantinib also appears to have some activity. A study of 112 patients with nccRCC included 17 with tRCC. The ORR in tRCC was 29% (5/17) with a median time to treatment failure of 8.3 months (95% *CI* 4·6–NR). The 12-month OS was 69% (95% *CI* 36–87%).

Given the largely retrospective nature of the published data, there is no clear evidence-based first-line therapy for these patients. The published data does suggest some activity for many of the TKI therapies with very limited activity with checkpoint inhibitors. In addition, given that there are very few patients treated with immune therapy published in the literature, it is suggested that TKI therapy be used first.

### Unclassified

Unclassified renal cell carcinoma (uRCC) comprises a heterogeneous mixture of kidney cancer that does not meet the diagnostic criteria of any other diagnosis. These cancers comprise diverse molecular alterations such as *NF2*, *SETD2*, and *mTOR* [44]. The prognosis appears to be poor relative to all other types of RCC. In 2247 patients undergoing partial or complete nephrectomy for localized RCC, the estimated 5-year OS for uRCC was more unfavorable compared to chRCC, PRCC, ccRCC, and clear cell papillary. The multivariate Cox model estimated the hazard ratio (HR) for OS of uRCC relative to low-grade conventional ccRCC as 2.58 (95% CI 1.34–4.95), in contrast, high-grade conventional ccRCC was only 1.42 (HR 0.87–2.30) [45].

Prospective studies specifically in uRCC are lacking. These patients have been included in some basket prospective trials including Keynote-427 cohort B [13•]. In total, 26 patients were treated with pembrolizumab. The ORR was 30.8% (95% *CI*, 14.3–51.8%) with a median PFS of 2.8 months (95% *CI*, 2.8–5.1), and the median OS was 17.6 months (95% *CI*, 7.5–NR).

Similarly, prospective trials with TKI therapy are also lacking. Some basket prospective trials have enrolled these patients as well as retrospective studies. The basket phase II trial of sunitinib for nccRCC from MD Anderson Cancer Center included 8 patients with uRCC [26•]. The ORR was 13% with a median PFS of 3.2 months (95% *CI* 1.4–NA). The multi-institution retrospective study of cabozantinib for nccRCC published by Harshman et al. included 15 patients with uRCC. The ORR was 13% (2/15) with an estimated median time to treatment failure of 6.0 months (95% *CI* 1.4–9.9 months). The 12-month OS was only 36% [9].

Further investigation into these molecular drivers will certainly better classify uRCC in the future into different, distinct diagnosis. It is encouraging that TKI and immune therapy appear clinically active for many of these patients.

### Sarcomatoid Differentiation

Sarcomatoid differentiation is not a true histologic classification and can be seen with any histology of RCC [46]. It is a positive predictive marker of response to immune therapy suggesting that regardless of the histologic diagnosis, immune therapy should be prioritized early in treatment for that patient [13•, 47].

## Conclusion

Historically, nccRCC was approached in clinical trials as a single diagnostic entity. Recent research into the pathophysiology of these diseases has led to a more diverse diagnostic framework. More recent clinical trials are fortunately now selecting patients based on specific disease histology and conducting fewer all-inclusive basket trials. This has led to guideline recommendations for histology-specific treatments and improved clinical outcomes for patients. Currently, TKI and immune therapy approaches dominate the treatment landscape for these diseases with ongoing clinical trials expected to further expand the treatment options for patients. There are many other types of RCC in the current WHO classification system than discussed here. With additional research and clinical trials, it is anticipated that more advances will be made in the future for patients diagnosed with these rare cancers.
